# TEIDe Consortium as a model to move towards a personalized medicine approach for the prevention of cognitive impairment and dementia

**DOI:** 10.1002/alz70860_102752

**Published:** 2025-12-23

**Authors:** Irene Esteban‐Cornejo, Kirk I. Erickson, Juan Ángel Bellón, Dorthe Stensvold, Elisabeth Wenger, Mario Possenti, Barbara Ukropcová, Teresa Liu‐Ambrose

**Affiliations:** ^1^ Instituto de Investigación Biosanitaria (ibs.GRANADA), Granada, Spain; ^2^ Faculty of Sport Sciences, Sport and Health University Research Institute (iMUDS), University of Granada., Granada, Granada, Spain; ^3^ CIBER de Fisiopatología de la Obesidad y Nutrición (CIBEROBN), Instituto de Salud Carlos III, Granada, Andalucia, Spain; ^4^ AdventHealth Research Institute, Orlando, FL, USA; ^5^ Biomedical Research Institute of Málaga and Nanomedicine Platform (IBIMA BIONAND Platform), Malaga, Andalucia, Spain; ^6^ Norwegian University of Science and Technology, Department of Circulation and Medical Imaging, Faculty of Medicine and Health Sciences, Trondheim, Norway, Norway; ^7^ Health and Medical University, Postdam., Postdam, Germany, Germany; ^8^ Federazione Alzheimer Italia, Milan, Milan, Italy; ^9^ Biomedical Research Center, Slovak Academy of Sciences, Bratislava, Slovakia; ^10^ Vancouver Coastal Health Research Institute, Vancouver, BC, Canada; ^11^ Centre for Aging SMART, Vancouver Coastal Health Research Institute, Vancouver, BC, Canada; ^12^ University of British Columbia, Vancouver, BC, Canada; ^13^ Djavad Mowafaghian Centre for Brain Health, Vancouver, BC, Canada

## Abstract

Dementia is a major cause of disability worldwide. Accurate identification of individuals at high risk of dementia is crucial for early diagnosis and prevention. **TEIDe** will examine the interplay between exercise and cognitive aging to develop practical and personalized applications in healthcare settings. Key questions addressed by the Consortium include: (i) How can predictive scores be developed to identify individuals at risk of all‐cause and cause‐specific dementia for primary healthcare implementation?; (ii) What are the optimal exercise doses and types depending on individual characteristics such as biological sex, gender, age, education, and cognitive/functional status?; (iii) Could different types of exercise exert benefits to cognition through various mechanisms at cellular/molecular, brain and behavioral levels? (iv) Is it feasible to implement a holistic approach for dementia prevention within primary healthcare settings, involving screening, tailored exercise prescription, and in‐person exercise interventions?

The overall aim of this consortium is to establish an integrated framework, combining predictive, precision, mechanistic, and clinical application approaches for the effective prevention of dementia. We will leverage data from existing cohort studies with over 3 million people and from 8 rigorously conducted exercise‐based randomized controlled trials in middle‐aged and older‐aged adults with a range of cognitive function; subsequently, we will test the feasibility for their clinical application in primary healthcare. To achieve this, 9 partners from 8 countries (i.e., Spain, Norway, Germany, Italy, Slovakia, Romania, USA and Canada) along the entire supply chain value (i.e., academia, primary healthcare sector, enterprise and operational stakeholder) compose this consortium.

TEIDe will build long‐term, world‐class research capacity to better understand the role of exercise interventions for the prevention of cognitive impairment and dementia. This project will generate at least four outputs, which can be easily implemented in healthcare settings: (i) risk scores for dementia identification; (ii) algorithms for tailored exercise prescription; (iii) user‐friendly exercise manuals for prescription in primary healthcare settings; (iv) modifiable risk factors amenable by exercise interventions. These deliverables will directly benefit patients and clinicians by preventing the progression of age‐related cognitive decline and early stages of Alzheimer's disease, and by moving towards a personalized medicine approach via improved prediction of individual treatment benefits for at‐risk groups

